# Global, Regional, and National Burdens of Parkinson's Disease in Adults Aged 20–50 Years, 1990–2021: A Cross‐Sectional Study

**DOI:** 10.1002/hsr2.71326

**Published:** 2025-10-13

**Authors:** Linxue Shen, Haizhen Xu, Kuihua Wang, Xiaoping Cui, Jianxin Ye

**Affiliations:** ^1^ Department of Neurology Fuzong Clinical Medical College of Fujian Medical University Fuzhou Fujian China; ^2^ Department of Neurology 900th Hospital of PLA Joint Logistic Support Force Fuzhou Fujian China

**Keywords:** early‐onset Parkinson's disease, global burden of disease, health inequality

## Abstract

**Background and Aims:**

Early‐onset Parkinson's disease (EOPD), characterized by symptom manifestation between the ages of 20 and 50 years, imposes substantial clinical, psychosocial, and economic burdens. We evaluated the global, regional, and national burdens of EOPD from 1990 to 2021, emphasizing trends in incidence, prevalence, mortality, and disability‐adjusted life years (DALYs) and projected future trajectories to 2050.

**Methods:**

Using age‐standardized rates (ASRs) and absolute numbers from the Global Burden of Disease 2021 study across 204 countries, this analysis covers incidence, prevalence, deaths, and DALYs from 1990 to 2021. we analyzed temporal trends via Joinpoint regression. A decomposition analysis was used to quantify the contributions of aging, population growth, and epidemiological shifts. Bayesian Age‐Period‐Cohort modeling projected the future burden. Health inequalities were assessed using the intercept, slope, and concentration indices.

**Results:**

Globally, the incidence and prevalence of EOPD increased steadily, with ASRs reaching 2.35 and 14 per 100,000, respectively, by 2021. Males had a 1.5‐fold higher incidence rate than females. The 45–50 age group exhibited the highest burden (age‐standardized incidence rate: 8.98 per 100,000). Southeast Asia, East Asia, and Oceania had the highest regional burden (age‐standardized incidence rate: 3.61 per 100,000). The high‐middle sociodemographic index (SDI) regions showed the fastest incidence growth (estimated annual percentage change [EAPC]: 2.05). Global deaths declined (EAPC: −0.42), but DALYs increased in the low‐middle SDI regions (EAPC: 0.32). This decomposition was attributed to aging and population expansion. Health inequalities indicated that high SDI regions exhibited a high burden of incidence but a low burden of mortality.

**Conclusion:**

The escalating burden of EOPD necessitates policies targeting modifiable risks (e.g., pesticide regulation), equitable access to healthcare, and early diagnosis. Genetic studies, occupational safety measures, and global health collaborations are vital to mitigate future impacts.

## 1 Introduction

Parkinson's disease (PD) is a progressive neurodegenerative disorder characterized by motor symptoms, such as bradykinesia, resting tremors, rigidity, and postural instability, as well as non‐motor manifestations, including cognitive impairment, mood disorders, autonomic dysfunction, and sleep disturbances [1]. Pathologically, PD is characterized by the degeneration of dopaminergic neurons in the substantia nigra pars compacta and accumulation of α‐synuclein aggregates known as Lewy bodies [2]. While PD predominantly affects older adults, with the incidence increasing with age, approximately 5%–10% of individuals with PD are diagnosed before the age of 50 years, a condition referred to as early‐onset PD (EOPD) [3]. EOPD presents unique challenges, including a lengthy disease trajectory, increased likelihood of developing motor complications from prolonged dopaminergic therapy, and significant psychosocial and economic burdens because of its onset during peak productive years [4]. Johnson et al. [5] demonstrated that newly diagnosed patients with early‐onset PD accrued an incremental economic burden of US $7383 in the first year after diagnosis, comprising an additional US $4072 in direct medical costs and US $3311 in indirect work‐loss costs relative to matched controls. Chaudhuri et al. [6] further indicate that individuals with early‐onset Parkinson's incur a substantially greater lifetime economic burden—driven by earlier disease initiation, prolonged disease duration, and peak productive‐year disruption—when both direct and indirect costs are considered. The etiology of PD is multifactorial and involves complex interactions between genetic predisposition and environmental exposures [3, 7, 8]. Genetic mutations, particularly in *PRKN*, *PINK1*, and *LRRK2*, are commonly associated with EOPD [3, 7]. Environmental risk factors, including exposure to pesticides, heavy metals, and head trauma, have also been implicated in increasing the risk of PD [9]. Notably, some of these factors are modifiable, suggesting potential avenues for developing preventive strategies.

In recent years, epidemiological trends of PD have undergone significant shifts, with a marked increase in PD prevalence among younger adult populations [10, 11]. Data from the Global Burden of Disease (GBD) 2021 highlight PD as the fastest‐growing neurological disorder. While previous studies have defined EOPD as cases with symptom onset between 15 and 50 years of age [12], juvenile‐onset PD (manifesting before the age of 20 years) remains exceedingly rare. Therefore, the 20–50‐year age range may represent a more appropriate definition for EOPD [4, 13]. In this study, we focused on a targeted analysis of the 20–50‐year age group to address critical knowledge gaps specific to this demographic population. Understanding the global, regional, and national burdens of EOPD is crucial for several reasons. First, it aids in the allocation of healthcare resources and development of targeted interventions tailored to younger populations. Second, it highlights the need for policies addressing the unique challenges faced by individuals with EOPD, including employment support and mental health services. Third, it may also identify unmet needs specific to this population compared to older populations. Finally, identifying regional variations in EOPD prevalence and incidence can provide insights into potential environmental or genetic risk factors and inform future research and prevention strategies.

We used data from the GBD 2021 to analyze the incidence, prevalence, mortality, and disability‐adjusted life years (DALYs) associated with PD in adults aged 20–50 years between 1990 and 2021. By elucidating these trends, we aimed to inform public health strategies and contribute to the global understanding of EOPD.

## 2 Methods

### 2.1 Data Sources

In this study, we used data from the GBD 2021, a comprehensive epidemiological resource that provides standardized estimates of disease burden across 204 countries and territories. For adults aged 20–50 years, we extracted age‐specific data on PD, including incidence, prevalence, deaths, and DALYs between 1990 and 2021. Age‐standardized rates (ASRs) were recalculated using the 20–50‐year population as a reference to ensure comparability across regions and time periods. To investigate regional variations, we stratified analyzes according to two key classification systems. First, we categorized data by Socio‐Demographic Index (SDI), a composite measure of development status comprising five distinct tiers. Second, we organized results according to the seven super‐regions defined by the GBD study. Furthermore, to capture national‐level heterogeneity, we conducted country‐specific analyzes for all 204 countries and territories. This hierarchical analytical framework enabled us to identify both macro‐level patterns and localized variations in disease burden. Ethical approval with waiver of informed consent was obtained from the University of Washington Institutional Review Board for use of deidentified GBD data.

### 2.2 Study Design

We performed mixed cross‐sectional and longitudinal analyzes to

1. Quantify the global, regional, and national burdens of PD among adults aged 20–50 years.
2. Evaluate gender‐specific trends using Joinpoint regression and pyramid modeling.
3. Elucidate the roles of population size, aging, and epidemiological shifts in influencing disease burden from 1990 to 2021 using decomposition analysis.
4. Analyze health inequalities across SDI quintiles (low, low‐middle, middle, high‐middle, and high).
5. Project incidence, prevalence, and ASRs up to 2050 using Bayesian Age‐Period‐Cohort (BAPC) modeling.

### 2.3 Statistical Analyzes

We used world maps to present the rates and numbers of incidence, prevalence, deaths, DALYs, and Estimated Average Percentage Change (EAPC) in 204 countries and regions. Temporal trends in ASRs for incidence, prevalence, deaths, and DALYs were analyzed using a Joinpoint regression model [14]. The Joinpoint Regression Model identifies significant trend changes by fitting piecewise linear regressions, quantifying trend variation rates across distinct time periods, and computing the overall trend. gender‐stratified models were used to identify inflection points and calculate annual percentage changes (APCs) with 95% confidence intervals (CIs). A maximum of three Joinpoints were permitted to balance model flexibility and parsimony. The EAPC was computed to quantify annual ASRs trends globally, across seven GBD super‐regions, gender, age, and 204 countries. The rates were mapped to visualize geographic disparities.

The relative contributions of population aging, growth, and epidemiological changes to disease burden were disaggregated across the SDI quintiles and seven GBD super‐regions using decomposition analysis [15]. That quantifies the relative contributions of distinct factors to observed changes in a target metric. Its fundamental principle involves partitioning aggregate variation into interpretable component drivers to identify key determinants. Health inequalities refer to systematic disparities in health status, healthcare access, or health outcomes between different social groups. These differences are typically associated with socioeconomic position, demographic characteristics, geographic location, or other structural determinants, and are considered both avoidable and unfair. Health inequality analysis [16] constitutes a systematic research approach to identify the social determinants of these disparities and evaluate their preventability and inequity, thereby providing evidence for policy interventions. Absolute Inequality Slope Index: Derived from a linear regression of ASR against SDI (0–1 scale), with steeper slopes indicating larger disparities between low‐ and high‐SDI populations. Concentration Index: Measured socioeconomic inequality in disease burden, with positive values indicating a high burden among advantaged groups.

The BAPC model [17] was used to forecast incidence, prevalence, deaths, and DALY case numbers and ASRs through 2050. Age effects (biological aging), period effects (e.g., healthcare advancements), and cohort effects (birth‐year influences) were partitioned, with 95% uncertainty intervals (UIs) generated via Markov Chain Monte Carlo sampling.

### 2.4 Statistical Software

Statistical analyzes were performed using Stata/MP 17 (64‐bit) with the “conindex” package for concentration indices and R 4.4.1 for all other analyzes. Key R packages included “dplyr” for data manipulation, “ggplot2” for visualization, and “MASS” for regression modeling. A two‐sided *p*‐value < 0.05 was considered statistically significant.

## 3 Results

### 3.1 Global Burden

**Figure 1:**
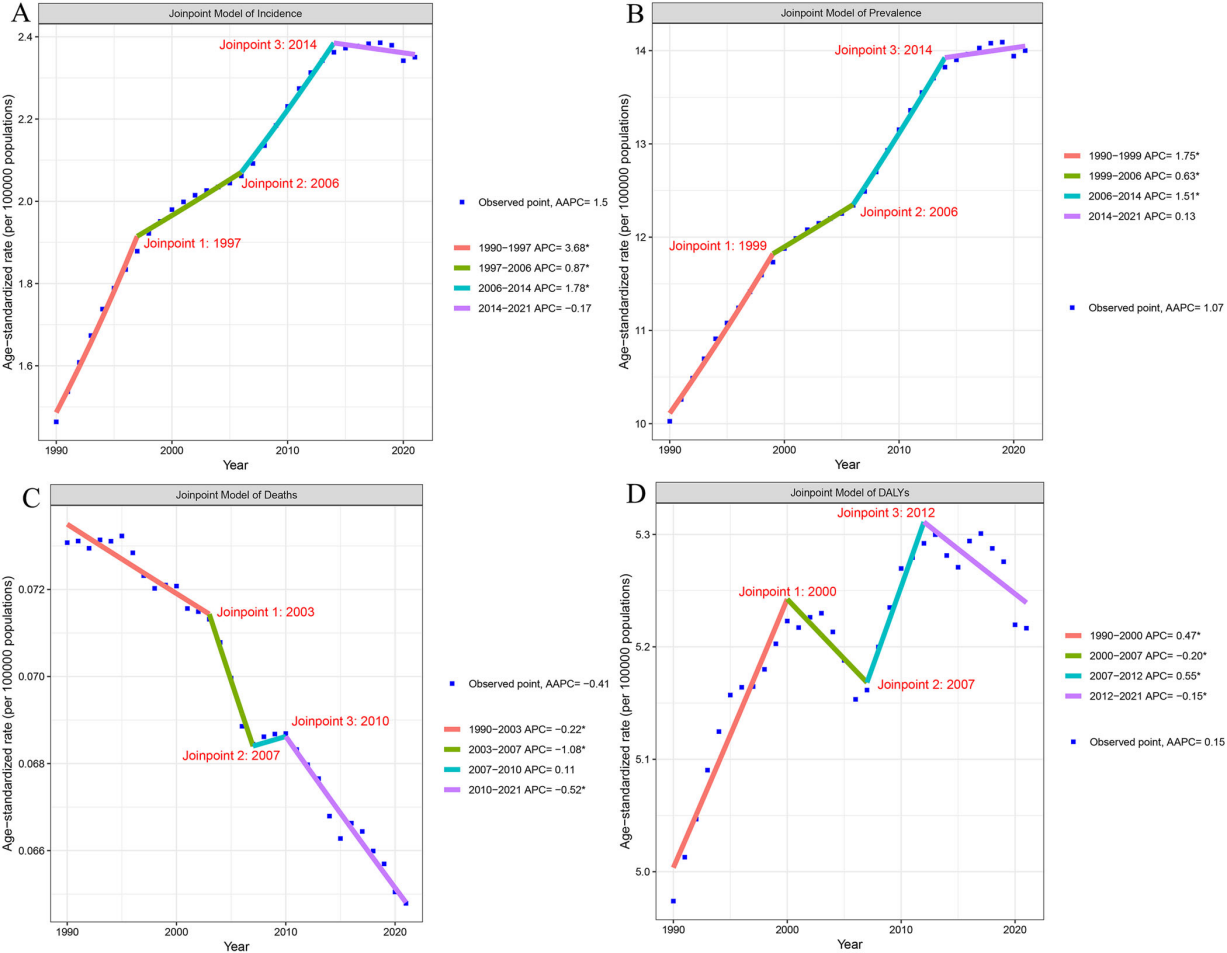
Joinpoint of early‐onset Parkinson's disease. (A) Changes over time based on the incidence model; (B) changes over time based on the prevalence model; (C) changes over time based on the deaths model; (D) changes over time based on the DALYs model. AAPC, average annual percent change; APC, annual percent change; DALYs, disability‐adjusted life‐years.

**Figure 2:**
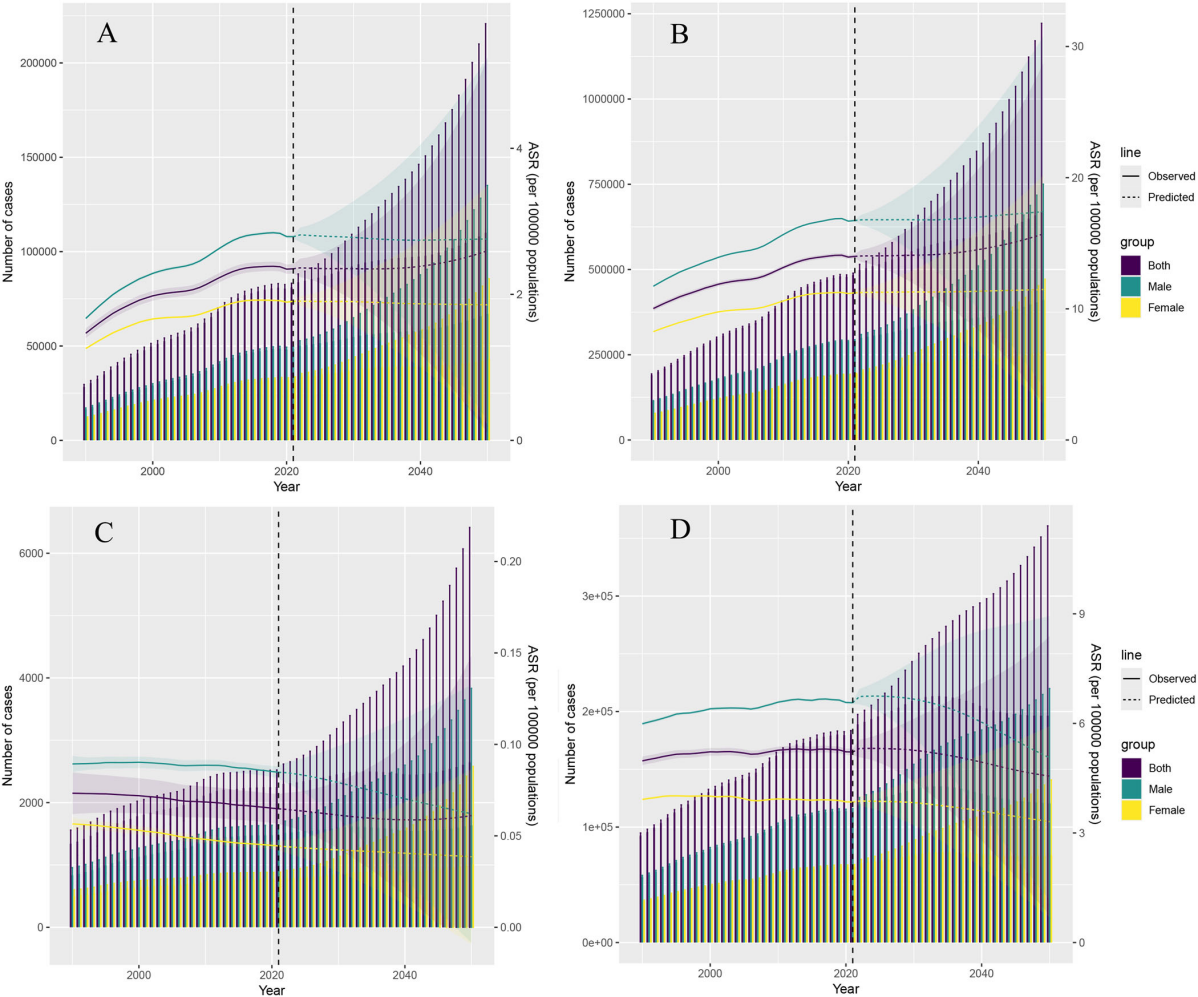
BAPC of early‐onset Parkinson's disease. (A) Incidence; (B) Prevalence; (C) deaths; (D) DALYs. ASR, age‐standardized rate; BAPC, Bayesian Age‐Period‐Cohort; DALYs, disability‐adjusted life‐years.

Globally, the incidence and prevalence of PD among adults aged 20–50 years have shown a continuous upward trend and are projected to continue to increase (Figures 1 and 2), with a more pronounced increase in prevalence than in incidence. The EAPC of incidence and prevalence from 2019 to 2021 were 1.42 (95% CI: 1.26–1.58) and 1.09 (95% CI: 1.02–1.16), respectively (Table 1). After 2014, the age‐standardized incidence rate (ASIR) declined (APC: −0.17), and the age‐standardized prevalence rate (ASPR) also decreased (APC: 0.13) (Figure 1). As of 2021, the global ASIR, ASPR, age‐standardized deaths rate (ASDR), and DALYs (ASR‐DALYs) in this age group were 2.35 per 100,000 (95% UI: 1.39–3.55), 14 per 100,000 (95% UI: 9.49–19.79), 0.06 per 100,000 (95% UI: 0.06–0.07), and 5.22 per 100,000 (95% UI: 4.22–6.52), respectively (Tables 1 and 2). The ASIR and ASPR in this age group are expected to continue to increase (Figure 2). By 2050, the ASIR is projected to be approximately 2.59 per 100,000 (95% CI: 0.11–2.53), with an estimated number of cases reaching 110,029.28 (95% UI: 4879.79–220,771.03). The ASPR is expected to rise by 12% compared with 2021, reaching approximately 15.71 per 100,000 (95% CI: 2.75–28.74), while the number of prevalent cases will increase by 38% to 669,653.10 per 100,000 (95% CI: 119,433.98–1,222,012.94) (Figure 2). From 1990 to 2021, the ASDR in this age group showed an overall downward trend (EAPC: − 0.42), whereas ASR‐DALYs exhibited a fluctuating upward trend (Figure 1). Similar to the overall population, the ASIR and ASPR in males were higher than those in females. In 2021, the ASIR in males was approximately 1.5 times higher than that in females (2.79 vs. 1.90), and the ASPR was approximately 1.2 times higher than that in females (1.18 vs. 0.98) (Table 1). Among the different age subgroups, the highest growth was observed in the 45–50 years age group; the ASIR was 8.98 per 100,000 in 2021 (95% CI: 5.49–13.4), with an EAPC of 1.88, and the ASPR was 56.18 per 100,000 (95% CI: 41.18–74.68), with an EAPC of 1.45 (Table 1). The growth in the ASIR and ASPR in the 40–44 years age group was concerning.

**Table 1:** The number and ASR of incidence and prevalence in 2021 and changing trends from 1990 to 2021.

	2021		1990–2021	2021		1990–2021
	Incidence cases (95% UI)	ASIR per 100,000 (95% UI)	EAPC of ASIR (95% CI)	Prevalence cases (95% UI)	ASPR per 100,000 (95% UI)	EAPC of ASPR (95% CI)
Global	81046.67 (48161.87–122328)	2.35 (1.39–3.55)	1.42 (1.26–1.58)	483872.47 (328861.91–682509.05)	14.00 (9.49–19.79)	1.09 (1.02–1.16)
Age						
20–24 years	812.19 (203.3–1583.54)	0.14 (0.03–0.27)	0.35 (0.3–0.39)	1378.07 (344.98–2678.19)	0.23 (0.06–0.45)	0.35 (0.3–0.39)
25–29 years	2383.28 (595.13–4629.52)	0.41 (0.1–0.79)	0.31 (0.27–0.35)	9275.32 (2317.62–18022.94)	1.58 (0.39–3.06)	0.31 (0.27–0.35)
30–34 years	5515.86 (3415.25–8029.88)	0.91 (0.56–1.33)	0.56 (0.54–0.59)	27958.95 (13220.81–47769.55)	4.63 (2.19–7.9)	0.39 (0.35–0.42)
35–39 years	9323.67 (3735.92–16564.28)	1.66 (0.67–2.95)	0.78 (0.75–0.8)	61405.57 (38042.04–89011.02)	10.95 (6.78–15.87)	0.6 (0.58–0.63)
40–44 years	20478.25 (14231.2–28053.98)	4.09 (2.84–5.61)	1.34 (1.15–1.53)	117862.7 (79942.1–171391.33)	23.56 (15.98–34.26)	0.9 (0.84–0.95)
45–49 years	42533.42 (25981.08–63466.8)	8.98 (5.49–13.4)	1.88 (1.63–2.14)	265991.86 (194994.36–353636.02)	56.18 (41.18–74.68)	1.45 (1.32–1.59)
Sex						
Male	48417.29 (28943.52–72816.64)	2.79 (1.67–4.2)	1.58 (1.42–1.75)	291072.89 (199491.69–405673.45)	16.74 (11.44–23.36)	1.18 (1.11–1.26)
Female	32629.38 (19138.04–50081.59)	1.90 (1.12–2.93)	1.21 (1.05–1.36)	192799.59 (129140.43–277911.64)	11.23 (7.49–16.23)	0.98 (0.9–1.05)
SDI						
High	8405.65 (5068.04–12457.87)	1.65 (0.98–2.45)	0.69 (0.65–0.74)	52827.07 (36616.17–73198.05)	10.26 (7.04–14.31)	0.36 (0.3–0.43)
High‐middle	20352.09 (12304.12–30496.68)	3.02 (1.82–4.55)	2.05 (1.79–2.32)	106896.06 (72767.31–152703.5)	15.78 (10.65–22.69)	1.6 (1.45–1.74)
Middle	32573.99 (19657.32–48825.4)	2.81 (1.69–4.21)	1.58 (1.35–1.8)	188369.30 (128754.69–263874.49)	16.15 (10.99–22.7)	1.26 (1.13–1.38)
Low‐middle	14599.23 (8292.46–22866.02)	1.88 (1.08–2.95)	0.83 (0.79–0.86)	99358.01 (65198.11–143977.93)	12.95 (8.56–18.69)	0.7 (0.67–0.74)
Low	5073.36 (2647.32–8137.05)	1.43 (0.76–2.29)	0.37 (0.32–0.42)	36135.64 (22909.84–53200.24)	10.50 (6.77–15.31)	0.35 (0.3–0.4)
GBD region						
High‐income	6987.43 (4048.62–10698.05)	1.43 (0.82–2.2)	0.26 (0.21–0.31)	48616.20 (33579.35–68496.74)	9.85 (6.72–14.01)	0.19 (0.1–0.27)
Southeast Asia, East Asia, and Oceania	39699.47 (24381.89–58878.15)	3.61 (2.21–5.37)	2.13 (1.76–2.5)	199665.89 (137040.33–280771.35)	18.05 (12.3–25.52)	1.7 (1.48–1.93)
South Asia	15224.84 (8373.3–23964.95)	1.95 (1.08–3.06)	0.92 (0.87–0.98)	107011.67 (69536.23–153542.11)	13.81 (9.03–19.76)	0.82 (0.76–0.87)
Central Europe, Eastern Europe, and Central Asia	2438.61 (1245.81–3894.05)	1.20 (0.61–1.93)	−0.11 (−0.17 to −0.05)	15617.42 (9970.43–23018.67)	7.61 (4.81–11.29)	−0.18 (−0.25 to −0.11)
North Africa and Middle East	5121.91 (2964.67–8055.55)	1.81 (1.05–2.84)	0.94 (0.9–0.97)	32135.51 (20412.83–47582.72)	11.37 (7.24–16.78)	0.88 (0.86–0.9)
Latin America and Caribbean	7330.65 (4405.82–11065.76)	2.72 (1.63–4.11)	0.91 (0.87–0.95)	49388.41 (33359.33–70501.8)	18.26 (12.32–26.09)	0.72 (0.68–0.77)
Sub‐Saharan Africa	4243.76 (2091.76–6988.49)	1.19 (0.59–1.94)	0.17 (0.13–0.22)	31437.38 (19506.81–46989.64)	9.10 (5.76–13.44)	0.18 (0.13–0.22)

**Table 2:** The number and ASR of deaths and DALYs in 2021 and changing trends from 1990 to 2021.

	2021		1990–2021	2021		1990–2021
	Deaths cases (95% UI)	ASDR per 100,000 (95% UI)	EAPC of ASDR (95% CI)	DALYs cases (95% UI)	ASR‐DALY per 100,000 (95% UI)	EAPC of ASR‐DALY (95% CI)
Global	2245.68 (1995.24–2495.67)	0.06 (0.06–0.07)	−0.42 (−0.45 to −0.39)	180325.32 (145990.3–225031.04)	5.22 (4.22–6.52)	0.14 (0.1–0.18)
Age						
20–24 years	14.09 (12.76–15.78)	0.00 (0.00–0.00)	−1.03 (−1.09 to −0.97)	1184.41 (993.28–1427.12)	0.2 (0.17–0.24)	−0.8 (−0.85 to −0.76)
25–29 years	21.50 (19.29–24.36)	0.00 (0.00–0.00)	−0.5 (−0.61 to −0.4)	2904.74 (1757.4–4409.71)	0.49 (0.3–0.75)	−0.09 (−0.13 to −0.05)
30–34 years	42.77 (39.02–47.71)	0.01 (0.01–0.01)	−0.67 (−0.82 to −0.53)	7158.21 (4495.26–10755.41)	1.18 (0.74–1.78)	−0.01 (−0.05 to 0.03)
35–39 years	64.69 (58.25–70.99)	0.01 (0.01–0.01)	−0.54 (−0.64 to −0.45)	13700.15 (8976.07–19941.06)	2.44 (1.6–3.56)	0.28 (0.25–0.3)
40–44 years	698.23 (613.49–793.54)	0.14 (0.12–0.16)	−0.39 (−0.46 to −0.33)	52904.56 (43535.29–64618.64)	10.58 (8.7–12.92)	0.02 (−0.03 to 0.06)
45–49 years	1404.4 (1252.43–1543.29)	0.30 (0.26–0.33)	−0.41 (−0.47 to −0.36)	102473.25 (86233–123879.11)	21.64 (18.21–26.16)	0.22 (0.15–0.29)
Sex						
Male	1475.44 (1277.38–1685.51)	0.08 (0.07–0.1)	−0.16 (−0.2 to −0.13)	114178.43 (91723.03–141330.67)	6.57 (5.27–8.13)	0.31 (0.27–0.35)
Female	770.24 (625.27–934.27)	0.04 (0.04–0.05)	−0.85 (−0.89 to −0.81)	66146.90 (51896.51–85190.92)	3.85 (3.01–4.96)	−0.11 (−0.14 to −0.08)
SDI						
High	275.29 (258.78–292.71)	0.05 (0.05–0.06)	0.2 (0.14–0.27)	20988.81 (17483.62–25486.68)	4.02 (3.33–4.91)	0.25 (0.22–0.29)
High‐middle	399.76 (344.69–471.76)	0.06 (0.05–0.07)	−1.07 (−1.18 to −0.96)	35210.86 (27709.48–46132.08)	5.21 (4.09–6.84)	−0.05 (−0.14 to 0.04)
Middle	829.82 (724.68–947.27)	0.07 (0.06–0.08)	−0.79 (−0.85 to −0.74)	67961.50 (54799.33–85364.52)	5.83 (4.69–7.34)	−0.04 (−0.09 to 0)
Low‐middle	520.46 (442.67–601.54)	0.07 (0.06–0.08)	0.09 (0.05–0.13)	40011.35 (31991–50316.11)	5.25 (4.21–6.59)	0.32 (0.29–0.34)
Low	218.69 (169.81–270.07)	0.07 (0.05–0.08)	−0.32 (−0.38 to −0.26)	16030.26 (12206.05–20541.65)	4.75 (3.64–6.05)	−0.08 (−0.13 to −0.04)
GBD region						
High‐income	220.88 (216–225.82)	0.04 (0.04–0.04)	0.03 (−0.05 to 0.11)	17851.89 (14667.89–22152.87)	3.53 (2.88–4.42)	0.08 (0.03–0.13)
Southeast Asia, East Asia, and Oceania	881.49 (741.98–1054.77)	0.08 (0.07–0.1)	−1.13 (−1.19 to −1.08)	71754.93 (57226.53–90970.8)	6.54 (5.21–8.31)	−0.17 (−0.23 to −0.11)
South Asia	460.29 (376.44–550.43)	0.06 (0.05–0.07)	0.09 (0.05–0.13)	38478.66 (29497.95–49444.15)	5 (3.85–6.41)	0.39 (0.36–0.43)
Central Europe, Eastern Europe, and Central Asia	85.10 (78.99–91.1)	0.04 (0.04–0.04)	−0.56 (−0.82 to −0.3)	6457.09 (5246.06–8150.59)	3.15 (2.55–3.98)	−0.42 (−0.57 to −0.27)
North Africa and Middle East	209.43 (169.72–251.18)	0.08 (0.06–0.09)	−0.58 (−0.65 to −0.5)	14679.28 (11756.62–18457.62)	5.24 (4.21–6.58)	−0.14 (−0.21 to −0.07)
Latin America and Caribbean	163.56 (150.77–178.37)	0.06 (0.06–0.07)	−0.16 (−0.2 to −0.11)	15447.43 (12107.22–20044.96)	5.71 (4.47–7.41)	0.27 (0.24–0.29)
Sub‐Saharan Africa	224.94 (167.51–302.04)	0.07 (0.05–0.09)	−0.07 (−0.11 to −0.04)	15656.04 (11610.93–21076.65)	4.64 (3.47–6.2)	0.01 (−0.01 to 0.03)

### 3.2 Regional and National Burdens

From the perspective of the SDI level, the ASIR was the highest and grew fastest in the high‐middle SDI regions, with an ASIR of 3.02 per 100,000 in 2021 (95% CI: 1.82–4.55) and an EAPC of 2.05 (Table 1). As of 2021, the ASPR was the highest in the middle SDI regions at 16.15 (95% CI: 10.99–22.7), but the growth rate was the fastest in the high‐middle SDI regions, with an EAPC of 1.60 (Table 1). The ASDR was the lowest in the high SDI regions at 0.05 per 100,000 (95% CI: 0.05–0.06), but the growth rate was the fastest at 0.2 (Table 2). The ASR‐DALYs were the highest in the middle SDI regions at 5.83 per 100,000 (95% CI: 4.69–7.34), whereas the growth rate was the fastest in the low‐middle SDI regions at 0.32 (Table 2).

At the regional level, the ASIR and ASPR were the highest in Southeast Asia, East Asia, and Oceania; the ASIR was 3.61 per 100,000 (95% CI: 2.21–5.37), with an EAPC of 2.13, whereas the ASPR was 18.05 per 100,000 (95% CI: 12.3–25.52), with an EAPC of 1.70 (Table 1). From 1990 to 2021, the ASDR in most GBD regions showed a downward trend, with only slight increases in high‐income (EAPC: 0.03) and South Asian (EAPC: 0.09) regions (Table 2). The ASR‐DALYs increased significantly in South Asia (EAPC: 0.39), Latin America, and the Caribbean (EAPC: 0.27) (Table 2).

**Figure 3:**
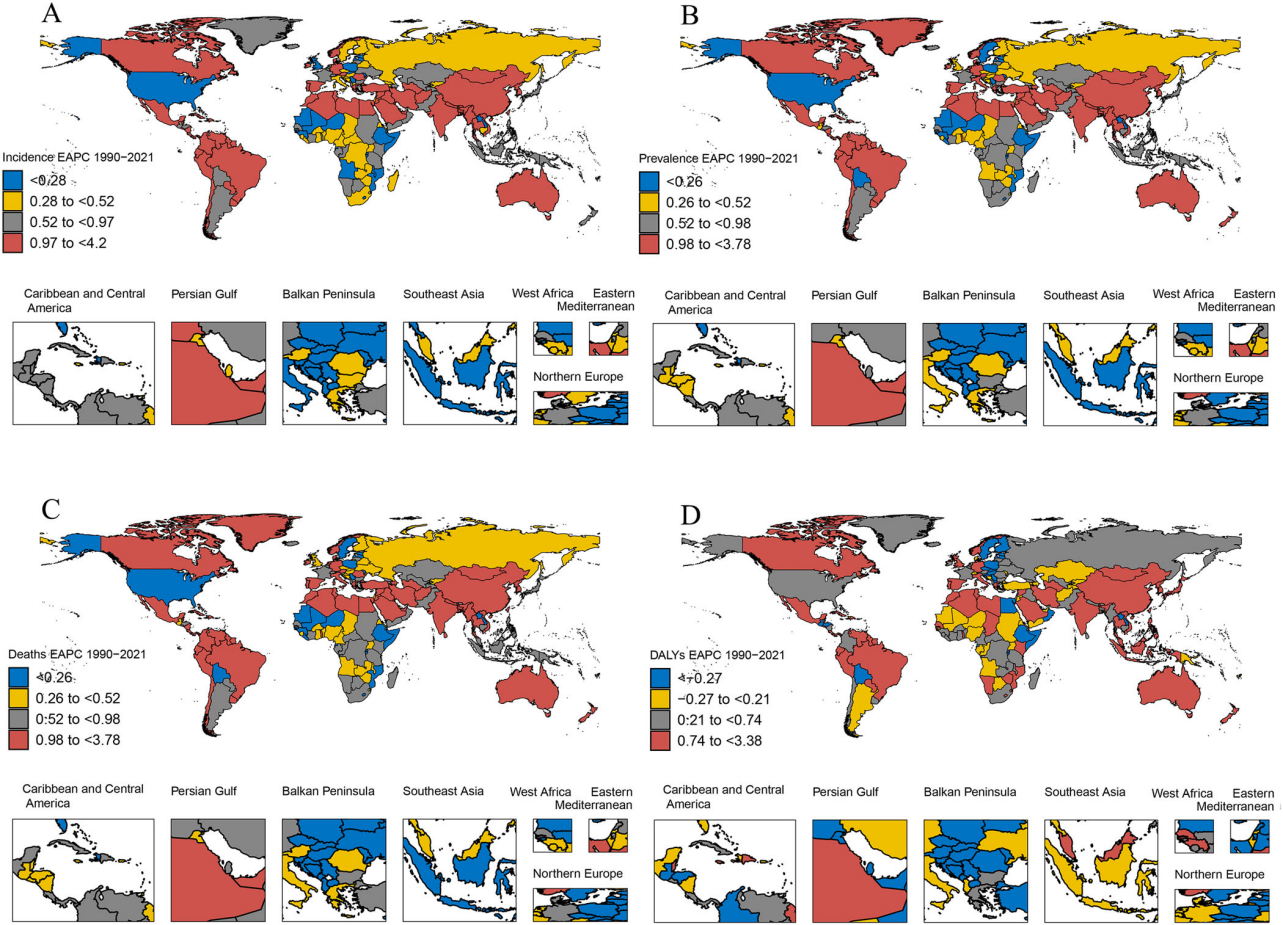
Global distribution maps of EAPC from early‐onset Parkinson's disease. (A) The incidence in terms of the EAPC from 1990 to 2021; (B) the prevalence in terms of the EAPC from 1990 to 2021; (C) the deaths in terms of the EAPC from 1990 to 2021; (D) the DALYs in terms of the EAPC from 1990 to 2021. DALYs, disability‐adjusted life‐years; EAPC, estimated average percentage change.

At the national level, there were significant differences in the burden of EOPD between countries. China had the highest burden, whereas Australia had the lowest. As of 2021, the incidence, prevalence, deaths, and DALYs in terms of both number and rate were in the highest burden category in China, and the EAPC of incidence, prevalence, deaths, and DALYs from 1990 to 2021 also grew at the fastest rate (Figures 3 and Supporting Information: Figures S1–S4). Canada and Brazil had a high burden of incidence and prevalence but a relatively low burden of deaths and DALYs (Supporting Information: Figures S1–S4). However, the EAPC of incidence, prevalence, deaths, and DALYs from 1990 to 2021 also increased at a relatively fast rate (Figure 3). In 2021, the ASIR and ASPR in the United States were low; however, the ASDR and ASR‐DALYs were moderate, and the EAPC of DALYs from 1990 to 2021 grew faster than that of the other three measures (Figure 3, Supporting Information: Figures S1 and S3). Overall, Australia had the lowest burden of EOPD, but the EAPC of incidence, prevalence, deaths, and DALYs increased rapidly from 1990 to 2021 (Figures 3 and Supporting Information: Figures S1–S4).

### 3.3 Decomposition Analysis

**Figure 4:**
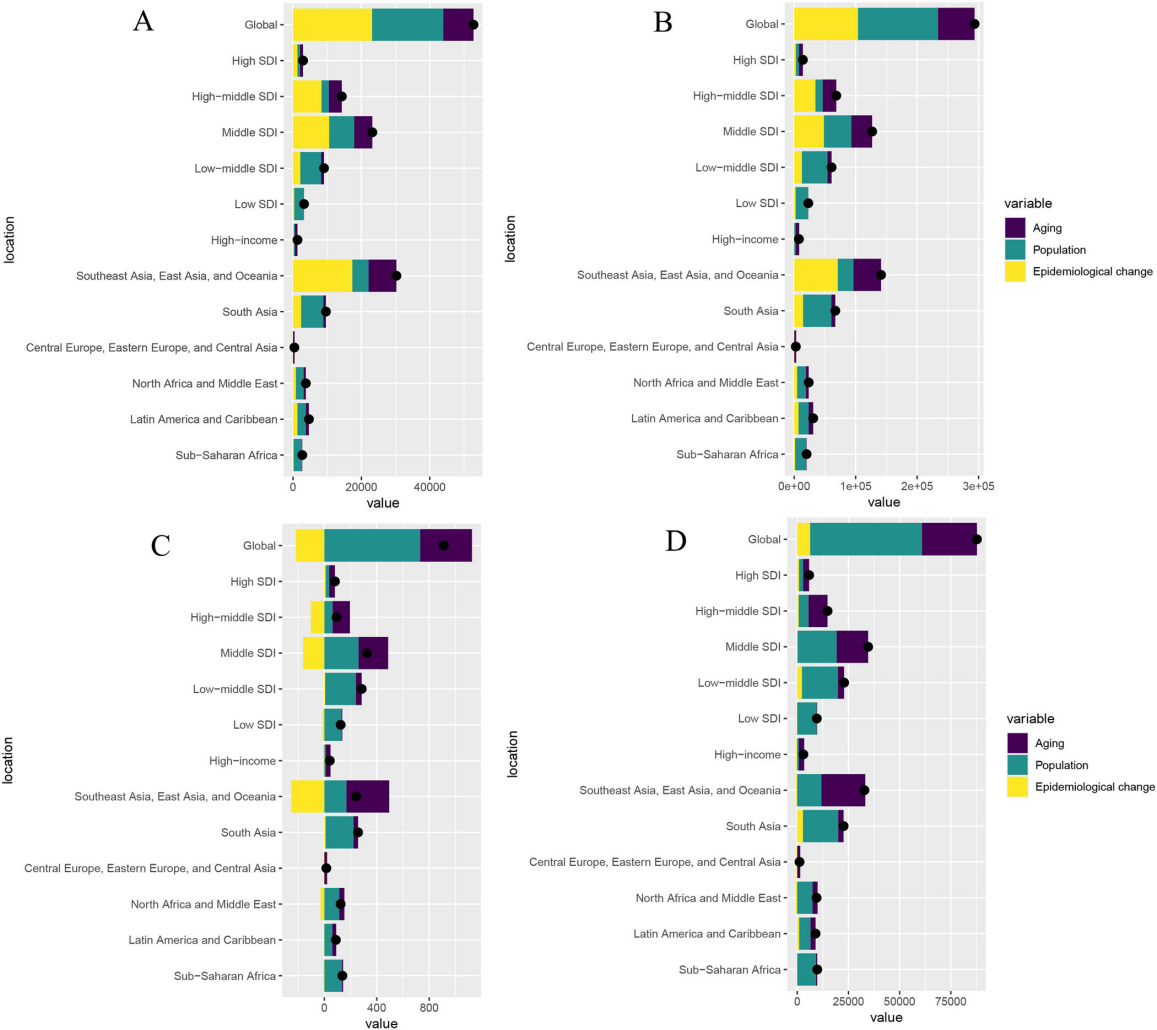
The decomposition of early‐onset Parkinson's disease. Changes in the burden of EOPD according to population‐level determinants (population growth, aging, and epidemiological changes) from 1990 to 2021 at the global level based on five SDI quintiles. (A) Incidence; (B) Prevalence; (C) Deaths; (D) DALYs. In all panels, the black dots represent the overall value of the change based on the contribution of all three components. DALYs, disability‐adjusted life‐years; EOPD, early‐onset Parkinson's disease; GBD, global burden of disease; SDI, socio‐demographic index.

A decomposition analysis was performed to examine changes in the number of EOPD cases across different locations between 1990 and 2021. The analysis considered several key factors, including aging, population growth, and epidemiological changes, and assessed their impact on incidence, prevalence, DALYs, and deaths (Figure 4 and Supporting Information: Table S1). Globally, aging, population growth, and epidemiological changes had positive impacts on incidence (Figure 4), with percentage changes of 16.82%, 39.35%, and 43.83%, respectively (Supporting Information: Table S1). These factors also positively affected the prevalence and DALYs (Figure 4), with percentage changes of 20.34%, 44.34%, and 35.32% for prevalence and 30.63%, 62.19%, and 7.18% for DALYs, respectively (Supporting Information: Table S1). Aging and population growth had positive impacts on deaths, whereas epidemiological changes had a negative impact (Figure 4), with percentage changes of 43.39%, 80.37%, and −23.76%, respectively (Supporting Information: Table S1).

In high‐middle SDI regions, aging and population growth had positive impacts on prevalence, whereas epidemiological changes had a negative impact (Figure 4), with percentage changes of 143.37%, 67.09%, and −110.46%, respectively (Supporting Information Table S1). Similar patterns were observed in the middle and low SDI regions. Except for the high SDI regions, the positive impact of aging on DALYs increased with high SDI levels, whereas the positive impact of population growth on DALYs decreased (Figure 4). Thus, as the SDI increased, the risk of incidence and prevalence owing to aging became significant, whereas the positive impact of population growth on mortality rates weakened. Additionally, in the high‐middle, middle, and low SDI regions, deaths declined over the past three decades owing to epidemiological changes in EOPD. These findings highlight the importance of considering aging, population growth, and epidemiological changes as key drivers of EOPD burden.

### 3.4 Health Inequality

**Figure 5:**
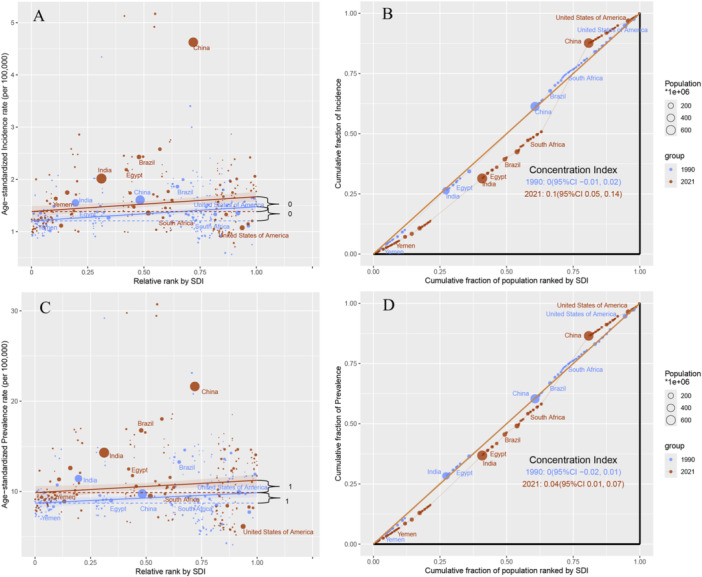
Health inequalities analysis of early‐onset Parkinson's disease. (A) Slope indices of inequality based on the incidence of EOPD in 1990 and 2021 (the numbers adjacent to the brackets indicate the slopes.); (B) concentration indices of inequality based on the incidence of EOPD in 1990 and 2021; (C) slope indices of inequality based on the prevalence of EOPD in 1990 and 2021; (D) concentration indices of inequality based on the prevalence of EOPD in 1990 and 2021; In these panels, each country or region is represented by a solid dot, with larger dots indicating a higher population. ASR, age‐standardized rate; EOPD, early‐onset Parkinson's disease; SDI, socio‐demographic Index.

The global health inequality analysis of EOPD is presented in Figure 5, Supporting Information: Figure 5 and Supporting Information: Table 2. Regarding incidence and prevalence, global absolute inequality increased, whereas relative inequality remained largely stable, with a tendency toward high incidence in regions with a high SDI (Figure 5). For incidence, the slope index expanded from 1.21 in 1990 (95% CI: 1.12–1.30) to 1.38 in 2021 (95% CI: 1.26–1.51), and the concentration index increased from 0.0035 in 1990 (95% CI: −0.0125 to 0.0194) to 0.0954 in 2021 (95% CI: 0.0539–0.1369) (Supporting Information: Table S2). The prevalence showed similar trends, but relative inequality also increased (Figure 5). The slope index expanded from 1.15 in 1990 (95% CI: 0.11–2.20) to 1.37 in 2021 (95% CI: −0.04 to 2.77), the intercept index increased from 8.72 in 1990 (95% CI: 8.07–9.36) to 9.88 in 2021 (95% CI: 8.99–10.77), and the concentration index increased from −0.0034 in 1990 (95% CI: −0.02 to 0.0131) to 0.0371 in 2021 (95% CI: 0.0085–0.0657) (Supporting Information: Table S2). The deaths showed the opposite trend. Absolute inequality remained largely stable, whereas relative inequality worsened, indicating a low death burden in regions with a high SDI (Figure 5). The slope index for death rates decreased from −0.03 in 1990 (95% CI: −0.04 to −0.02) to −0.04 in 2021 (95% CI: −0.05 to −0.03) (Supporting Information: Table S2). For DALYs, both absolute and relative inequalities worsened, with the burden decreasing as the SDI increased (Figure 5). The slope index for DALYs decreased from −1.29 in 1990 (95% CI: −1.72 to −0.86) to −1.78 in 2021 (95% CI: −2.27 to −1.29), and the intercept index increased from 5.14 in 1990 (95% CI: 4.87–5.40) to 5.40 in 2021 (95% CI: 5.09–5.70) (Supporting Information: Table S2).

## 4 Discussion

Our findings are consistent with those of previous studies and indicate a continuous increase in the global burden of EOPD. The results of our study confirmed that both the incidence and prevalence of EOPD have been increasing steadily from 1990 to 2021, with EAPC were 1.42 and 1.09, respectively. Projections indicate that these upward trends will persist through 2050. These findings are consistent with previous GBD reports, which have highlighted a general increase in PD cases across all age groups, albeit with regional variations [12, 18, 19, 20]. For instance, previous studies have shown that while the incidence and prevalence of PD are traditionally lower in East Asia than in Western countries, recent trends suggest a sharp increase in this region, with EAPCs of 2.13 vs −0.11 for incidence and 1.70 vs −0.18 for prevalence, respectively, potentially owing to industrialization and environmental exposure [21]. Additionally, we observed a higher incidence and prevalence of EOPD in males than in females, with men having an approximately 1.5 times higher incidence rate than women. This gender disparity has been widely documented in the literature, with hypotheses suggesting that estrogen may provide neuroprotection against dopaminergic neuron degeneration, potentially explaining the low prevalence of PD in females. Furthermore, occupational and environmental exposures, such as pesticide use and heavy metal exposure, have been implicated in the pathogenesis of PD and are more common among men than among women, which may contribute to the observed gender differences [22, 23, 24]. Regionally, our findings highlighted that the highest EOPD burden was observed in Southeast Asia, East Asia, and Oceania, where ASIR and ASPR peaked at 3.61 per 100,000 and 18.05 per 100,000, respectively. This regional disparity mirrors findings from previous research, which noted that the prevalence of PD is not uniformly distributed across the globe but is influenced by genetic [25, 26, 27, 28], environmental [29, 30, 31], and healthcare accessibility [32, 33, 34] factors. Additionally, while high‐income countries exhibit stable or even slightly declining mortality rates owing to advancements in PD management, middle‐ and low‐income countries continue to experience increasing DALYs because of limited healthcare resources and diagnostic challenges.

The increasing burden of EOPD poses significant challenges to healthcare systems worldwide, necessitating strategic policy responses to address its prevention, early detection, and management. Given the increasing incidence and prevalence rates of EOPD, governments and healthcare organizations should prioritize it as a public health concern. The World Health Organization has emphasized the need for a global action plan for neurological disorders, including PD, underscoring the importance of improving diagnostic and treatment strategies [35]. One critical policy consideration is environmental regulation, particularly concerning exposure to neurotoxicants, such as pesticides, industrial chemicals, and heavy metals. Previous studies have revealed a strong link between prolonged pesticide exposure and increased risk of PD [9, 36, 37, 38, 39]. Implementing strict regulations regarding the use of hazardous chemicals and promoting occupational safety measures can reduce the incidence of EOPD. Another crucial policy area is to improve access to early diagnosis and specialized care. Given that patients with EOPD are often in their prime working years, timely diagnosis and intervention are essential for maintaining their quality of life and economic productivity. Such productivity comprises wage employment, self‐employment, household production and informal caregiving; its disruption generates both direct medical costs and indirect work‐loss expenses—Johnson et al. [5] estimated an incremental first‐year burden of US $7383 per patient. Policymakers should invest in training healthcare professionals—particularly primary‐care physicians and other allied health professionals with relevant expertise—to consider younger‐onset presentations routinely and to facilitate prompt referrals to neurologists. Additionally, integrating telemedicine and artificial intelligence‐based diagnostic tools into routine clinical practice can enhance early detection, particularly in resource‐limited settings. Finally, disparities in access to healthcare must be addressed. Many low‐ and middle‐income countries lack infrastructure for adequate PD management, leading to delayed diagnoses and suboptimal treatment. Ensuring equitable access to essential medications, such as levodopa and dopamine agonizts, as well as surgical interventions, such as deep brain stimulation, should be a priority in global health initiatives. Establishing international funding mechanisms or pharmaceutical aid programs may help bridge these gaps and improve the outcomes of patients with EOPD.

As PD is traditionally viewed as an age‐related disorder, younger patients often experience diagnostic delays or misdiagnoses [1, 40, 41]. Educational campaigns targeting both medical professionals and the public could improve early recognition of symptoms, reduce the time to diagnosis, and enable earlier intervention [42]. Currently, most countries manage EOPD according to general treatment guidelines that include pharmacotherapy, surgical interventions, and rehabilitation programs. Only a few nations (e.g., China, the United States and the United Kingdom) have issued supplementary recommendations or consensus statements explicitly addressing EOPD‐specific considerations. However, owing to the unique challenges faced by younger patients, such as prolonged disease duration and high social and occupational impacts, additional tailored management strategies are required. A key strategy to improve disease management is the systematic implementation of national PD registries and guidelines. Such registries would allow for better epidemiological surveillance, resource allocation, and research on disease progression and treatment responses. Several high‐income countries have established PD databases. However, expanding these initiatives to low‐income regions can significantly improve global PD management. Therefore, it is essential to raise awareness among healthcare providers and the public of the need to consider a younger age range more routinely so that EOPD can be diagnosed and treated at an earlier stage. Multidisciplinary care approaches should also be emphasized. In those areas that have PD clinics, patients with EOPD benefit from integrated care teams that include neurologists, physical therapists, occupational therapists, and mental health professionals. Psychological support is particularly important, as younger patients with PD are likely to experience depression and anxiety related to their diagnoses. Encouraging lifestyle modifications, such as regular physical exercise, has been shown to have neuroprotective effects and should be incorporated into PD management programs.

Several factors likely contribute to the increased burden of EOPD worldwide. Improved diagnostic capabilities and increased awareness of PD among healthcare professionals are likely to contribute to the high reported incidence and prevalence rates. Advances in neuroimaging and biomarker research have facilitated early and accurate diagnoses, leading to improved case identification [43]. Environmental exposure plays a crucial role in the etiology of PD. Studies have linked chronic exposure to pesticides, industrial solvents, and air pollution to an increased PD risk. Regions undergoing rapid industrialization, such as East and South Asia, have reported significant increases in PD incidence, supporting the hypothesis that environmental risk factors are major contributors to disease burden [9, 36, 37, 38, 39]. Genetic predisposition is also a key factor. While most PD cases are diopathic, genetic mutations, particularly in *PARK2*, *PINK1*, and *LRRK2*, have been identified in a subset of patients with EOPD [2, 3, 7, 8]. Specific populations who carry these genes at high frequencies may exhibit disproportionately high rates of early‐onset cases. Further genetic and epidemiological studies are required to elucidate the role of hereditary factors in different geographical regions. Demographic changes also influence the epidemiology of PD. Although EOPD is defined as onset before age 50, demographic ageing and rising life expectancy have enlarged the population at risk. Global life expectancy at birth rose from 64.2 years in 1990 to 73.3 years in 2019 [44]. Additionally, in high‐income countries, improved healthcare access has led to frequent and early diagnoses, inflating the reported prevalence rates.

Despite the strengths of this study, it has several limitations. First, reliance on GBD data introduces potential biases owing to data availability and quality variations across countries. Many low‐income regions lack comprehensive neurological disease surveillance, leading to potential underreporting or misclassification of EOPD cases. Second, variations in the diagnostic criteria across studies may affect comparability. Although we defined EOPD as onset before the age of 50 years, some studies used a strict threshold (e.g., age 40 years), potentially leading to differences in the reported prevalence rates [11, 45, 46, 47]. Third, we focused primarily on epidemiological trends and did not include patient‐level data on genetic and environmental risk factors. Future studies incorporating multiomic data and longitudinal follow‐ups could provide deep insights into disease mechanisms. Finally, projections of future PD burden are based on current trends and may not fully account for potential breakthroughs in treatment or prevention strategies. Emerging therapies, such as gene editing and neuroprotective drugs, could significantly alter future PD epidemiology, underscoring the need for ongoing surveillance and modeling refinement.

Several areas warrant further investigation. In the future, genetic and environmental determinants of EOPD should be explored using large‐scale, multi‐ethnic cohort studies. Identifying population‐specific genetic risk factors and gene‐environment interactions is crucial for developing personalized prevention and treatment strategies. Longitudinal studies tracking disease progression in patients with EOPD may provide valuable insights into disease heterogeneity and treatment responses. Additionally, studies assessing the impact of public health interventions, such as pollution control policies and workplace safety regulations, on PD incidence could inform preventive strategies. Finally, the development of novel disease‐modifying therapies remains a priority. With advances in regenerative medicine, gene therapy and immunomodulatory approaches, future studies should focus on translating these innovations into effective, disease‐modifying treatments and improved diagnostic strategies for EOPD. Critically, these trials must incorporate the unique unmet needs of younger patients—including delayed diagnosis, earlier and more severe levodopa‐related motor complications, and disproportionate work‐loss and psychosocial burden—which differ from those encountered in late‐onset PD.

## 5 Conclusion

This study highlights the increasing global burden of EOPD and its implications for public health. Addressing this growing challenge requires a multifaceted approach involving improved surveillance, targeted prevention strategies, equitable access to healthcare, ongoing research on the underlying mechanisms of the disease, and novel therapies.

## Author Contributions

**Linxue Shen:** methodology, data curation, software, visualization, writing – original draft, writing – review and editing. **Haizhen Xu:** conceptualization, data curation, writing – original draft, writing – review and editing, methodology. **Kuihua Wang:** writing – original draft, writing – review and editing. **Xiaoping Cui:** methodology, conceptualization, project administration, writing – review and editing, writing – original draft, supervision. **Jianxin Ye:** conceptualization, methodology, project administration, writing – original draft, writing – review and editing, supervision, funding acquisition.

## Consent

All authors have read and approved the final version of the article. Jianxin Ye and Xiaoping Cui had full access to all of the data in this study and takes complete responsibility for the integrity of the data and the accuracy of the data analysis.

## Conflicts of Interest

The authors declare no conflicts of interest.

## Transparency Statement

The lead author Xiaoping Cui, Jianxin Ye affirms that this manuscript is an honest, accurate, and transparent account of the study being reported; that no important aspects of the study have been omitted; and that any discrepancies from the study as planned (and, if relevant, registered) have been explained.

## Supporting information


**Figure S1:** Global distribution maps of number from early‐onset Parkinson's disease. (A) the incidence in 2021; (B) the prevalence in 2021; (C) the deaths in 2021; (D) the DALYs in 2021. ASR, age‐standardized rate; DALYs, disability‐adjusted life‐years.

**Figure S2:** Global distribution maps of ASR from early‐onset Parkinson's disease. (A) the incidence in 2021; (B) the prevalence in 2021; (C) the deaths in 2021; (D) the DALYs in 2021. ASR, age‐standardized rate; DALYs, disability‐adjusted life‐years.

**Figure S3:** Global distribution maps of number from early‐onset Parkinson's disease. (A) the incidence in 1990; (B) the prevalence in 1990; (C) the deaths in 1990; (D) the DALYs in 1990. DALYs, disability‐adjusted life‐years.

**Figure S4:** Global distribution maps of ASR from early‐onset Parkinson's disease. (A) the incidence in 1990; (B) the prevalence in 1990; (C) the deaths in 1990; (D) the DALYs in 1990. ASR, age‐standardized rate; DALYs, disability‐adjusted life‐years.

**Figure S5:** Health inequalities analysis of early‐onset Parkinson's disease. (A) slope indices of inequality based on the deaths of EOPD in 1990 and 2021 (the numbers adjacent to the brackets indicate the slopes.); (B) concentration indices of inequality based on the deaths of EOPD in 1990 and 2021; (C) slope indices of inequality based on the DALYs of EOPD in 1990 and 2021; (D) concentration indices of inequality based on the DALYs of EOPD in 1990 and 2021; In these panels, each country or region is represented by a solid dot, with larger dots indicating a higher population. ASR, age‐standardized rate; EOPD, early‐onset Parkinson's disease; SDI, socio‐demographic Index.

**Table S1:** The decomposition of number for Parkinson's disease from 1990 to 2021.

**Table S2:** The slope, intercept, and concentration indicies of ASR for Parkinson's disease from 1990 to 2021.

## Data Availability

The data that support the findings of this study are available in Global Burden of Disease at http://www.healthdata.org. These data were derived from the following resources available in the public domain: GBD Results Tool, https://vizhub.healthdata.org/gbd-results/. The data that support the findings of this study are openly available in the Global Burden of Disease at https://vizhub.healthdata.org/gbd-results/.
