# Supplementary material for: Global, Regional, and National Burdens of Parkinson's Disease in Adults Aged 20–50 Years, 1990–2021: A Cross‐Sectional Study
Source: Health Sci Rep. 2025 Oct 13;8(10):e71326. doi: 10.1002/hsr2.71326 (PMC12516154; doi:10.1002/hsr2.71326)
Supplement: Supplementary file 1 — Figure S1: Global distribution maps of number from early‐onset Parkinson's disease. (A) the incidence in 2021; (B) the prevalence in 2021; (C) the deaths in 2021; (D) the DALYs in 2021. ASR, age‐standardized rate; DALYs, disability‐adjusted life‐years. [file HSR2-8-e71326-s006.pdf]

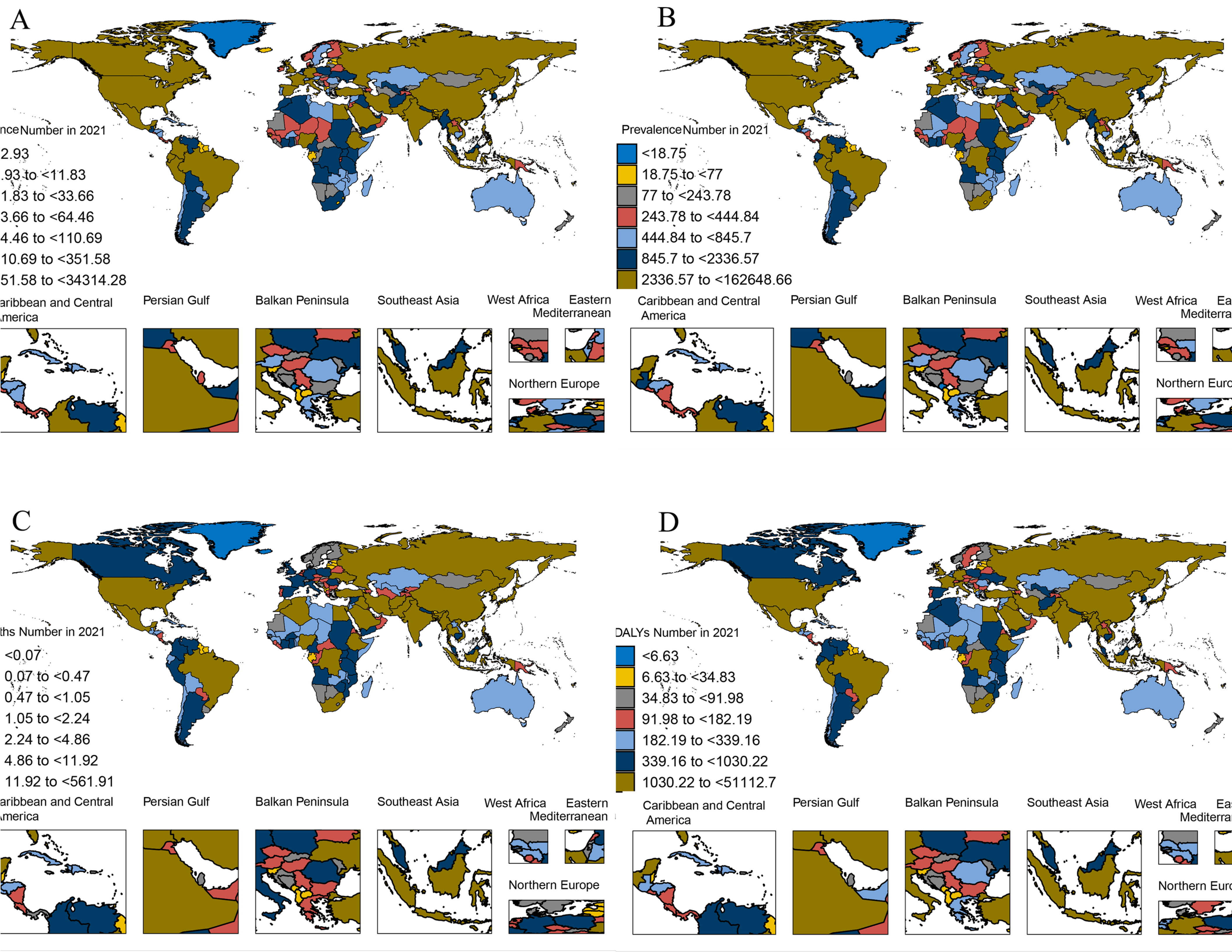
A
nce Number in 2021
2.93
.93 to <11.83
1.83 to <33.66
3.66 to <64.46
4.46 to <110.69
10.69 to <351.58
51.58 to <34314.28
aribbean and Central
merica
Persian Gulf
Balkan Peninsula
Southeast Asia
West Africa
Eastern
Mediterranean
Northern Europe
B
Prevalence Number in 2021
<18.75
18.75 to <77
77 to <243.78
243.78 to <444.84
444.84 to <845.7
845.7 to <2336.57
2336.57 to <162648.66
Caribbean and Central
America
Persian Gulf
Balkan Peninsula
Southeast Asia
West Africa
Ea
Mediterra
Northern Euro
C
ths Number in 2021
<0.07
0.07 to <0.47
0.47 to <1.05
1.05 to <2.24
2.24 to <4.86
4.86 to <11.92
11.92 to <561.91
aribbean and Central
merica
Persian Gulf
Balkan Peninsula
Southeast Asia
West Africa
Eastern
Mediterranean
Northern Europe
D
DALYs Number in 2021
<6.63
6.63 to <34.83
34.83 to <91.98
91.98 to <182.19
182.19 to <339.16
339.16 to <1030.22
1030.22 to <51112.7
Caribbean and Central
America
Persian Gulf
Balkan Peninsula
Southeast Asia
West Africa
Ea
Mediterra
Northern Euro
