# Supplementary figures and images for: Global, Regional, and National Burdens of Parkinson's Disease in Adults Aged 20–50 Years, 1990–2021: A Cross‐Sectional Study
Source: Health Sci Rep. 2025 Oct 13;8(10):e71326. doi: 10.1002/hsr2.71326 (PMC12516154; doi:10.1002/hsr2.71326)

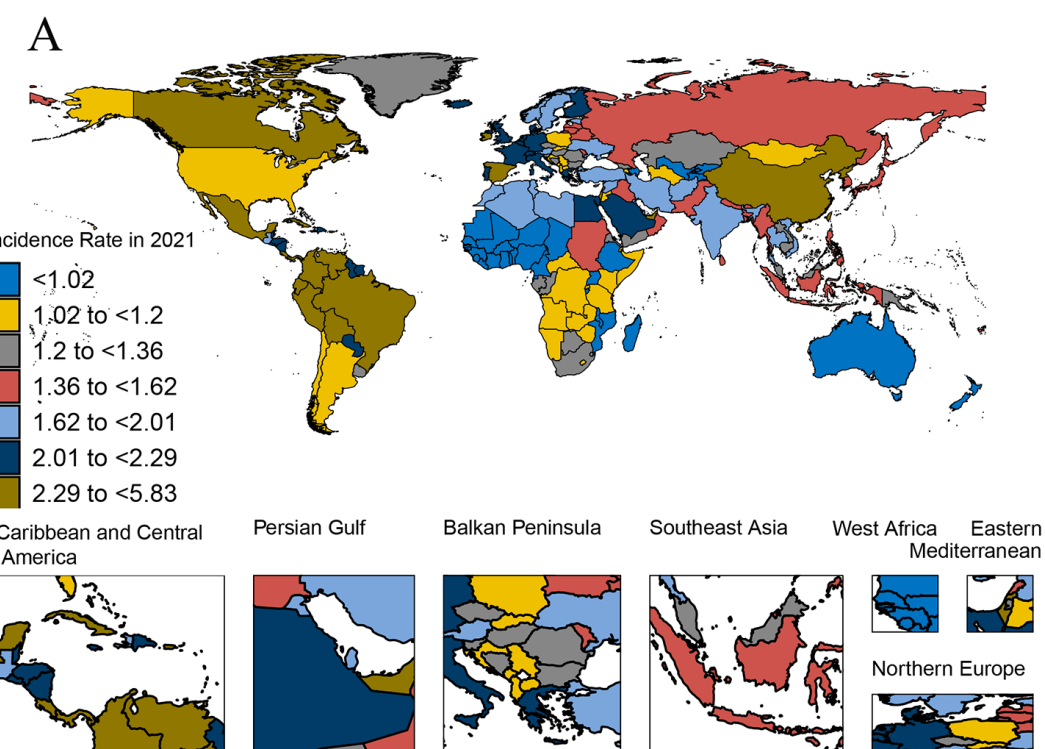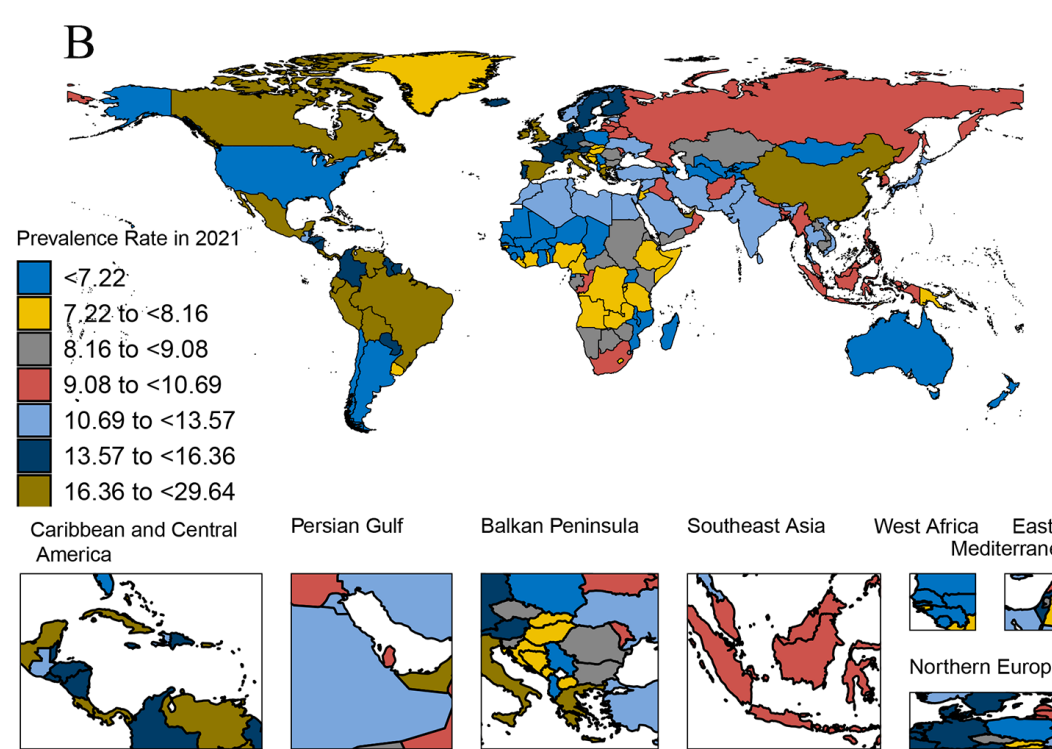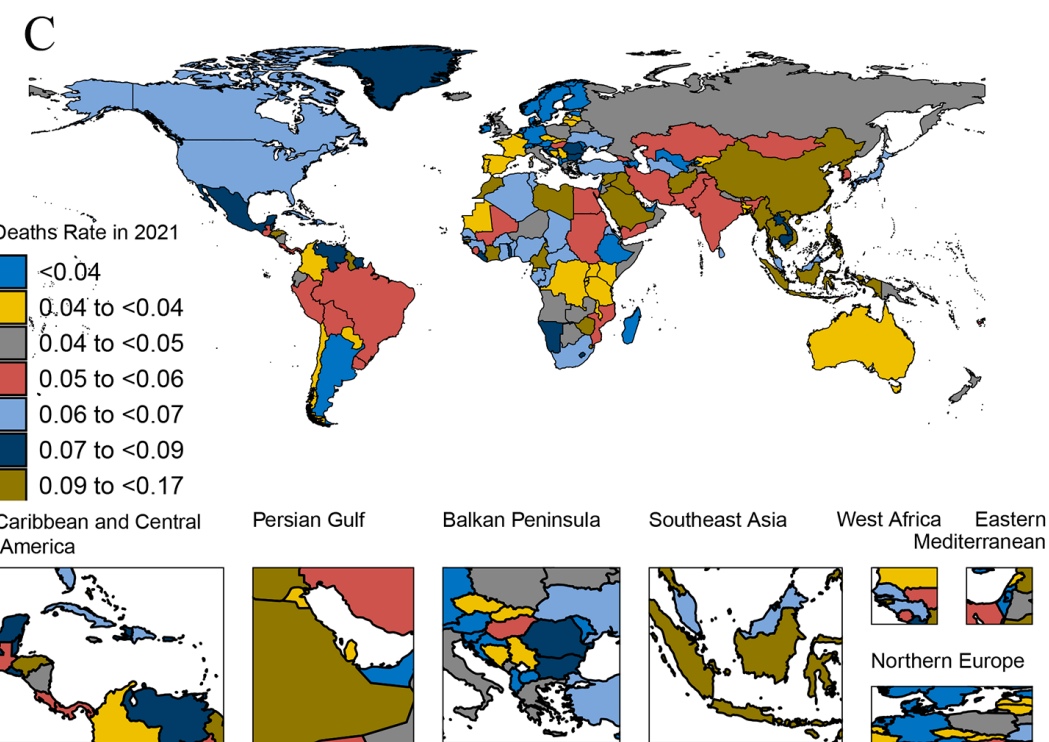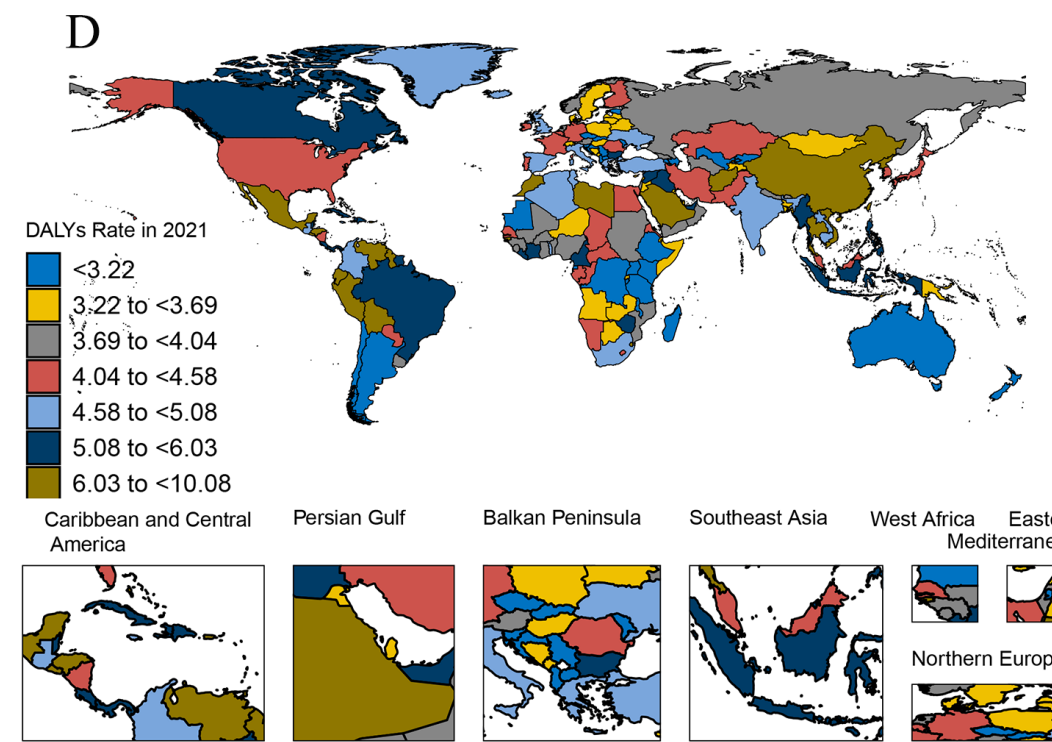

Supplement: Supplementary file 2 — Figure S2: Global distribution maps of ASR from early‐onset Parkinson's disease. (A) the incidence in 2021; (B) the prevalence in 2021; (C) the deaths in 2021; (D) the DALYs in 2021. ASR, age‐standardized rate; DALYs, disability‐adjusted life‐years. [file HSR2-8-e71326-s005.pdf]

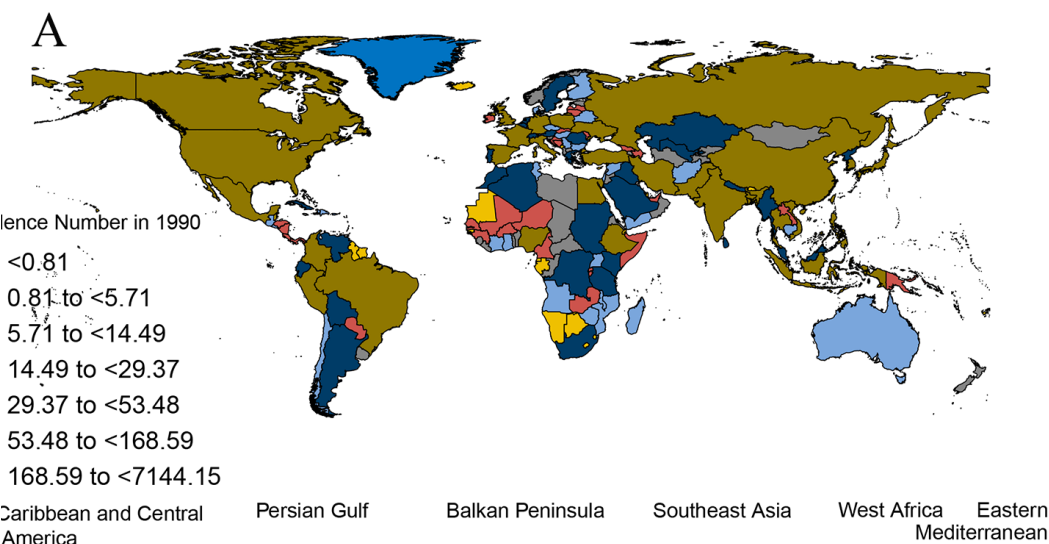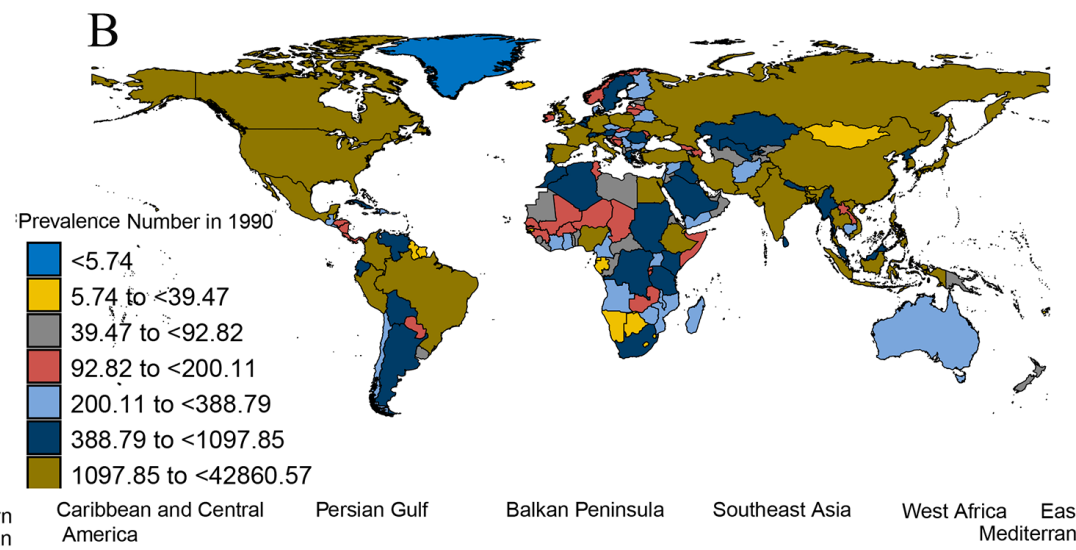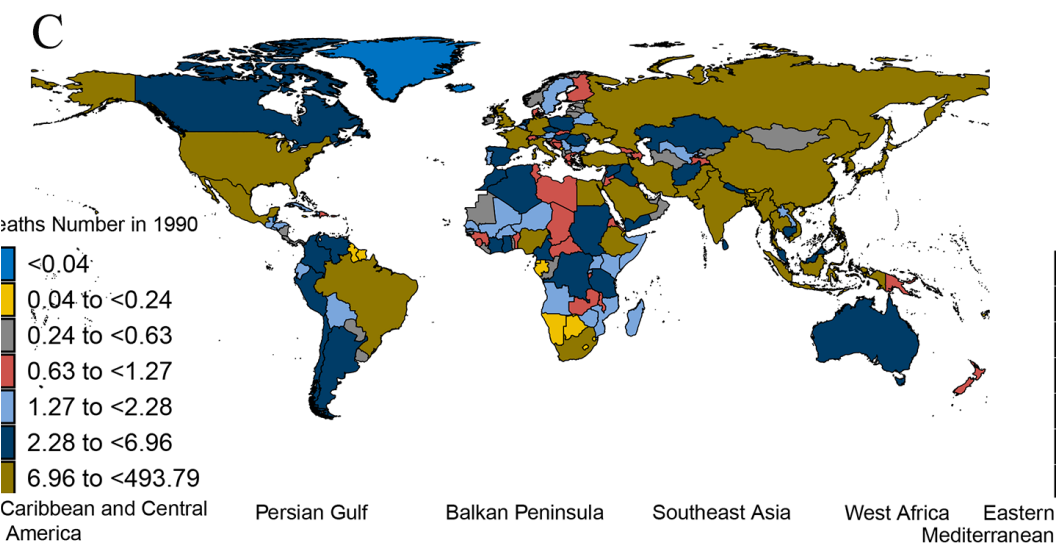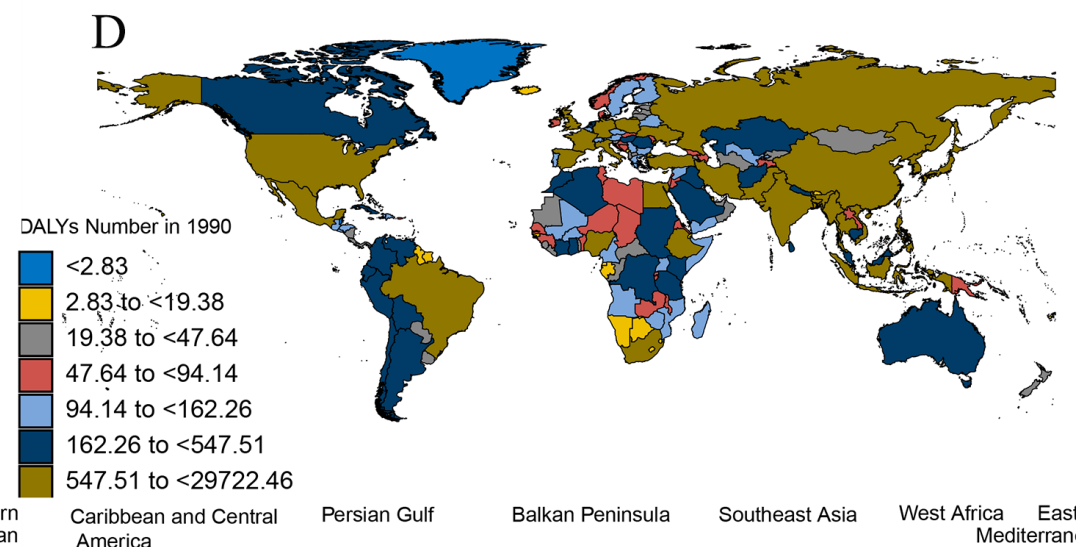

Supplement: Supplementary file 3 — Figure S3: Global distribution maps of number from early‐onset Parkinson's disease. (A) the incidence in 1990; (B) the prevalence in 1990; (C) the deaths in 1990; (D) the DALYs in 1990. DALYs, disability‐adjusted life‐years. [file HSR2-8-e71326-s001.pdf]

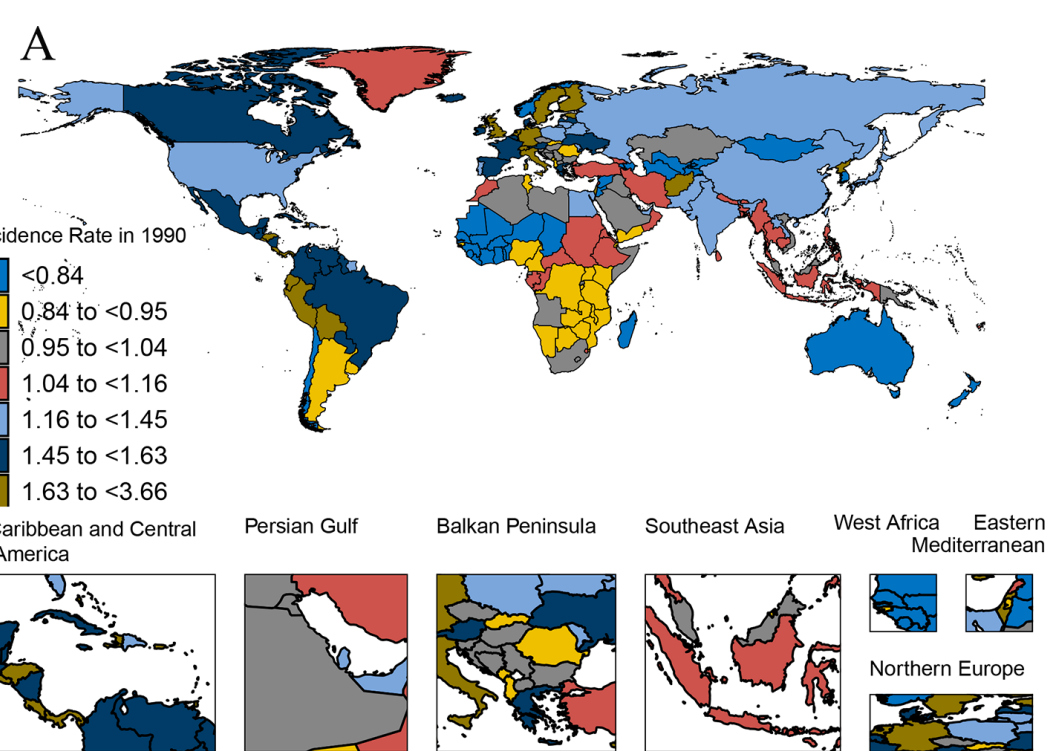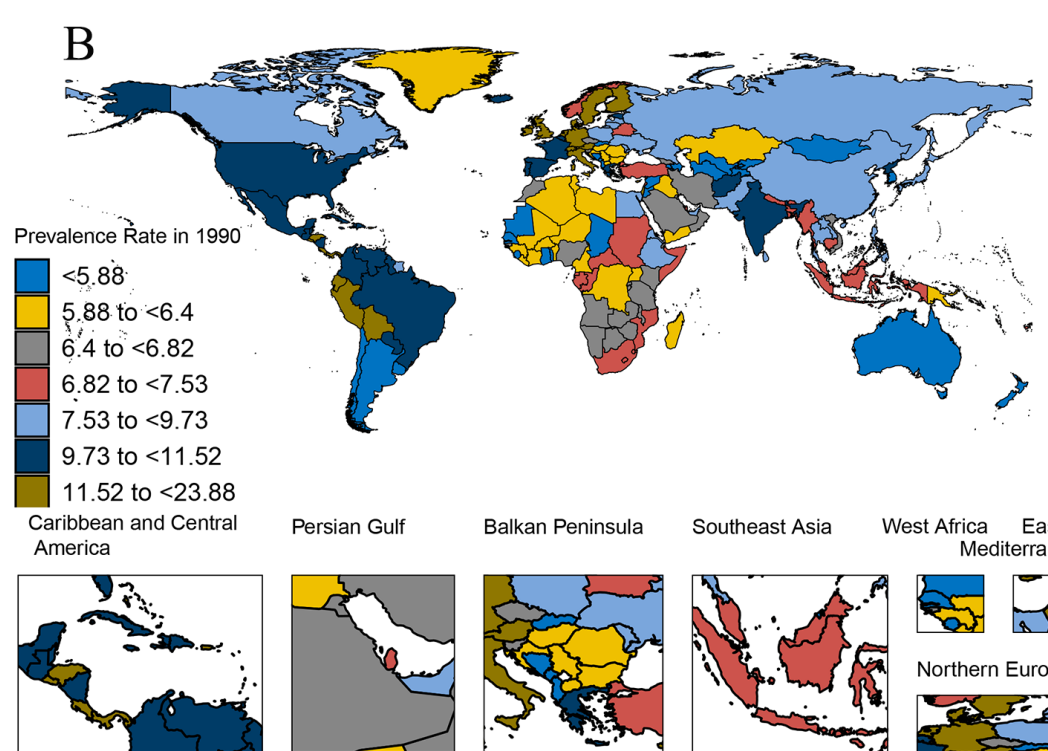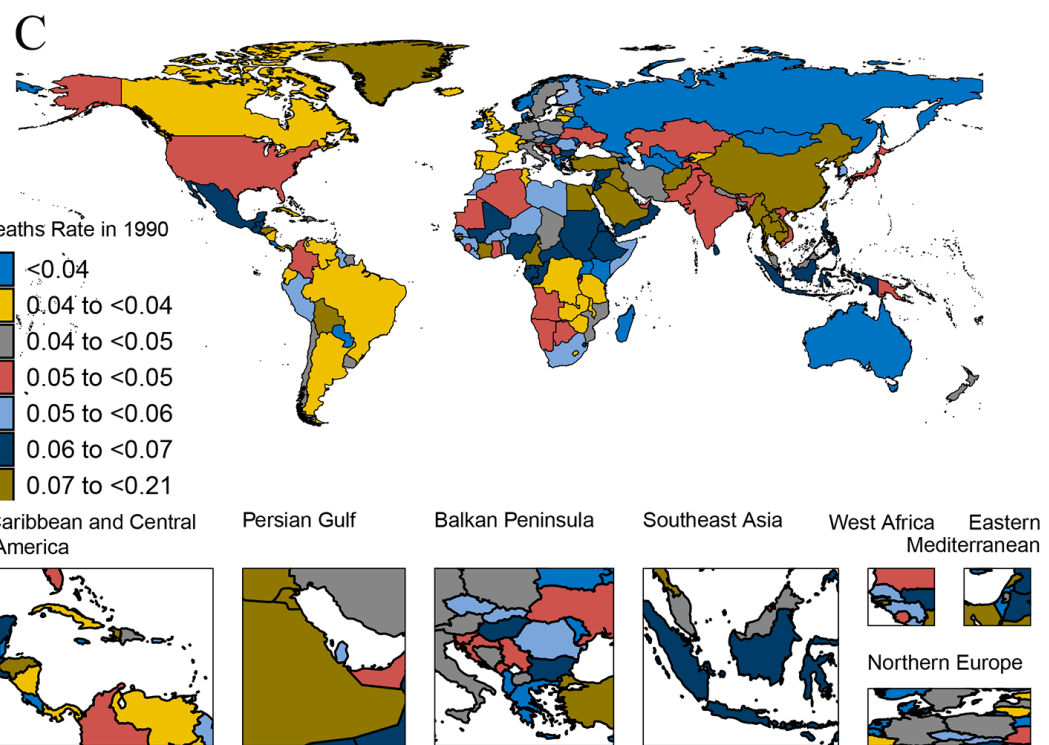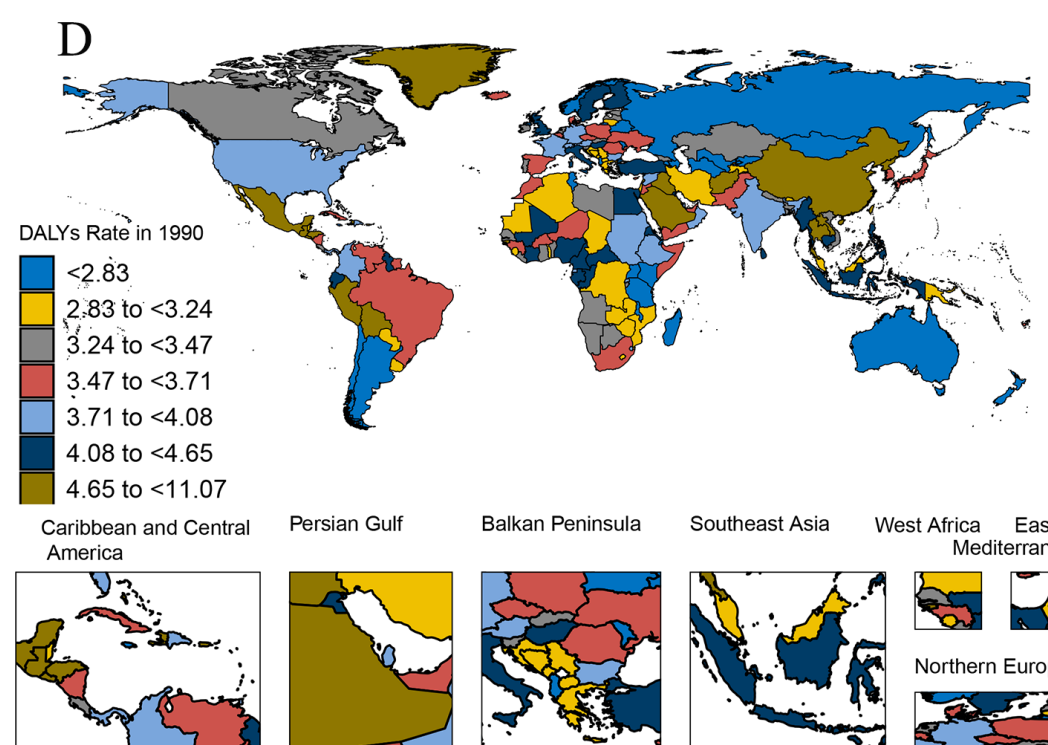

Supplement: Supplementary file 4 — Figure S4: Global distribution maps of ASR from early‐onset Parkinson's disease. (A) the incidence in 1990; (B) the prevalence in 1990; (C) the deaths in 1990; (D) the DALYs in 1990. ASR, age‐standardized rate; DALYs, disability‐adjusted life‐years. [file HSR2-8-e71326-s003.pdf]

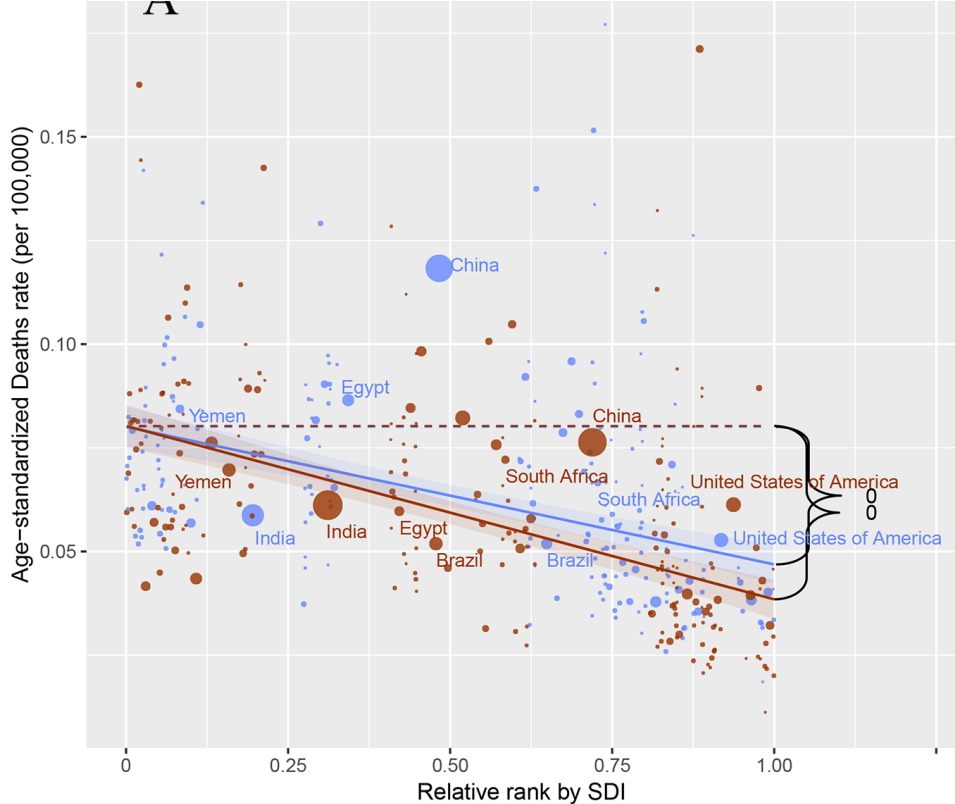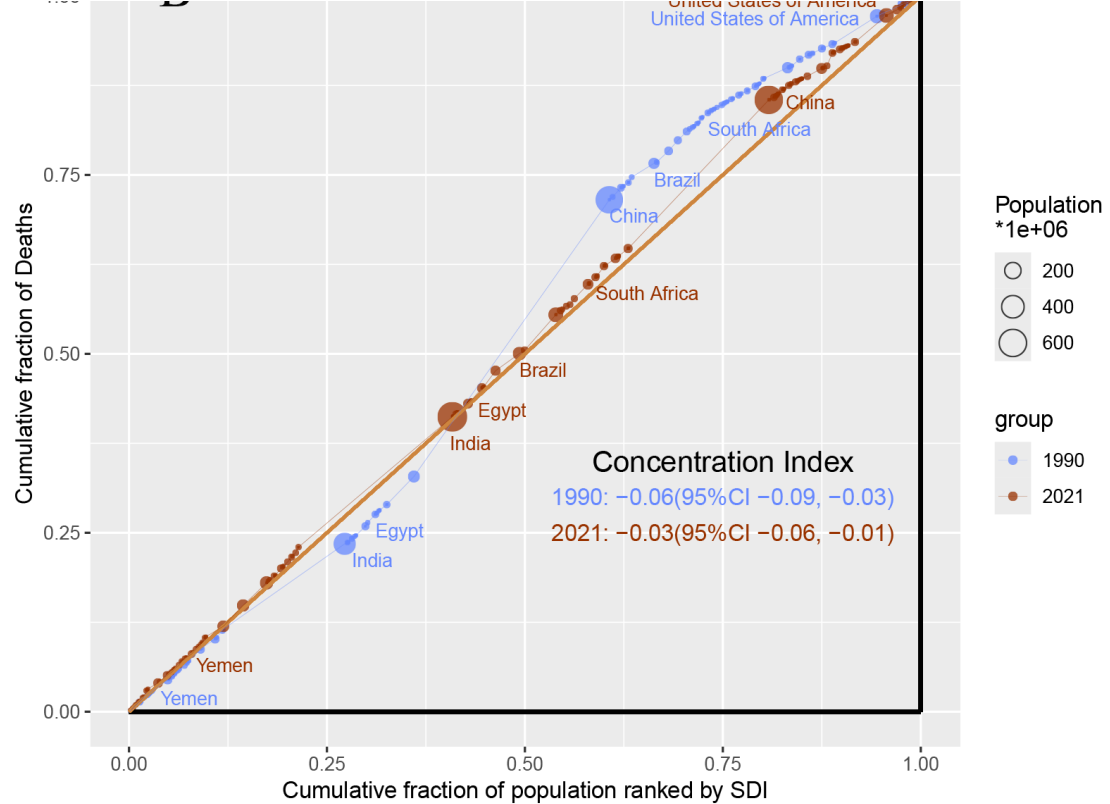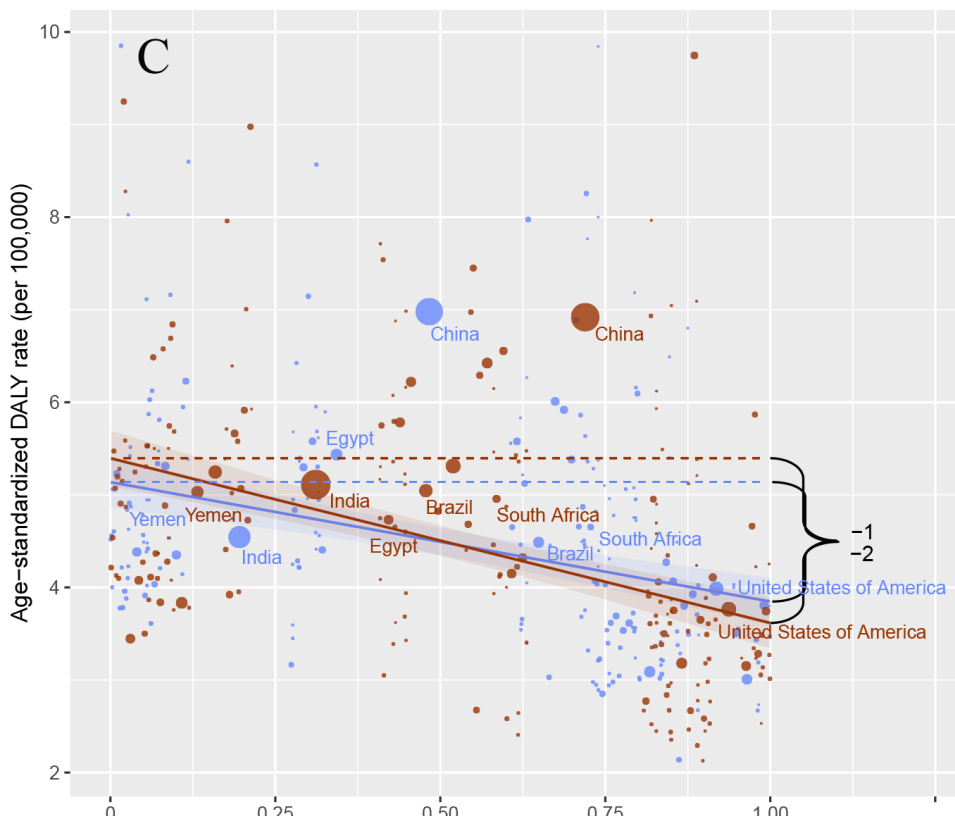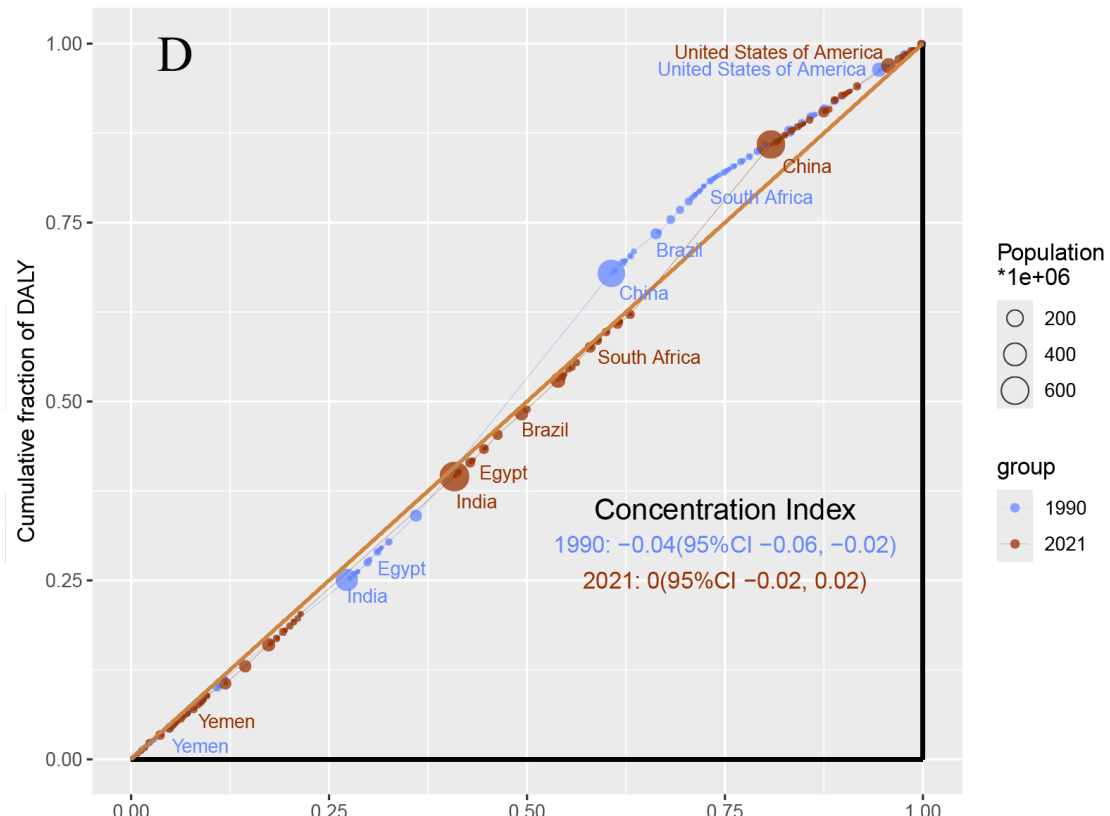

Supplement: Supplementary file 5 — Figure S5: Health inequalities analysis of early‐onset Parkinson's disease. (A) slope indices of inequality based on the deaths of EOPD in 1990 and 2021 (the numbers adjacent to the brackets indicate the slopes.); (B) concentration indices of inequality based on the deaths of EOPD in 1990 and 2021; (C) slope indices of inequality based on the DALYs of EOPD in 1990 and 2021; (D) concentration indices of inequality based on the DALYs of EOPD in 1990 and 2021; In these panels, each country or region is represented by a solid dot, with larger dots indicating a higher population. ASR, age‐standardized rate; EOPD, early‐onset Parkinson's disease; SDI, socio‐demographic Index. [file HSR2-8-e71326-s002.pdf]
